# Theoretical analysis of dynamic sliding mechanism of rock slope with a bedding structural plane based on stress wave propagation

**DOI:** 10.1038/s41598-024-72966-z

**Published:** 2025-02-28

**Authors:** Shaobo Chai, Boyang Song, Jinhao Liu, Kai Liu, Xinyuan Liu, Jiehui Shi

**Affiliations:** 1 School of Civil Engineering, Chang’an University, Xi’an, 710064 China; 2 Department of Engineering Science, University of Oxford, Parks Road, Oxford, OX1 3PJ UK

**Keywords:** Stress wave propagation, Bedding rock slope, Rock structural plane, Slip mechanism, Coefficient of stability, Civil engineering, Natural hazards

## Abstract

The stress at the structural plane of bedding rock slope will change under dynamic load, which may lead to sliding failure risk of the slope. Based on the time-domain recursive method (TDRM), the propagation process of stress waves in viscoelastic rock slope with a nonlinear bedding plane is analyzed, and the propagation equation of multiple reflected waves between the plane and the slope surface is obtained. According to the superposition principle and the relation between the particle vibration velocity caused by stress waves and stress, the expressions of normal and tangential stress of any particle at the structural plane are obtained. Furthermore, in light of the gravitational impact on the rock mass, we formulate the slip criterion equation for the structural plane. The results indicate that the stress field at the structural plane is influenced by several factors, including the slope angle, horizontal positioning of monitoring points, vertical distance to the slope foot, and the initial stiffness of the structural plane. The influence of multiple reflected waves on stress field obviously increases the possibility of rock mass sliding on structural plane. This paper theoretically elucidates the slippage mechanism of a rock slope featuring a bedding structural plane subjected to the effects of stress wave. The research findings furnish a theoretical foundation for comprehending the dynamic response of rock slopes, conducting dynamic stability analyses, and ensuring the safety measures for rock mass engineering projects.

## Introduction

Natural rock masses often contain numerous structural planes, leading to the discontinuity and nonuniformity of rock mass. These characteristics profoundly affect various engineering properties of the rock mass^1–6^. Blasting activities are frequently carried out in engineering excavations, resulting in the generation of impact loads that affect the rock mass and propagate as stress waves. When acting on the discontinuity, stress waves will interact with the discontinuity to produce transmitted and reflected waves. This interaction results in a complex wave field within the rock mass^7–10^. As each wave passes through the structural plane and reaches the ground surface, it causes vibrations on the surface. Consequently, the analysis of stress wave propagation within rock masses featuring structural planes provides the essential framework for understanding the vibrations resulting from stress wave propagation.

It is inevitable to construct a large number of complex artificial slopes in most rock mass engineering. The safety of rock slope under dynamic load will be a challenging problem during both the construction process and subsequent operation. It is evident that rock slope failures predominantly occur along the structural planes within the rock mass. The dynamic actions pose a significant threat to the stability of bedding rock slopes. Consequently, based on the interaction between structural plane and stress wave, the dynamic response of rock slope with structural plane under dynamic load is studied, and on this basis, the deformation and failure mechanism of slopes are thoroughly analyzed. Such research holds paramount theoretical importance in advancing our comprehension of the response mechanisms of rock mass under dynamic loads, forecasting and mitigating geological hazards related to rock masses, and providing valuable guidance for preliminary planning, construction design, and ongoing maintenance.

The current research on the dynamic response of rock slope is mainly carried out by two main power sources: blasting load and seismic load. For the research of slope vibration response caused by dynamic load, the traditional research methods are mainly physical test and numerical simulation. Singh et al.,^11^ studied the movement and deformation law of the overlying strata caused by underground mining and discussed the stability of mining operations on the dangerous rock mass on the surface by using numerical simulation combined with the field geological conditions. Azizabadi et al.,^12^ systematically analyzed the influence of blasting vibration on the stability of rock slope by using discrete element numerical simulation method and waveform superposition theory. Fan et al.,^13^ studied the dynamic response and failure mode of rock slope under dynamic load through shaking table test. Zhang et al.,^14^ used PFC2D to simulate the deformation mechanism and movement characteristics of landslide under earthquake action. The instability and failure of rock slope are closely related to the structural characteristics of the slope. The presence of rock structural planes significantly influences the dynamic response of rock slopes. Bedding and anti-inclined rock slopes exhibit distinct dynamic response characteristics and instability failure modes. Regarding the dynamic response of rock slopes with structural planes, numerous scholars have conducted valuable explorations and research, elucidating fundamental phenomena and patterns to a considerable extent^15,16^, but there is still room for further research and discussion within the field. At present, there is a lack of research concerning the dynamic response patterns and slope sliding mechanism in rock slopes with structural plane, particularly from the perspective of wave propagation characteristics.

When stress wave propagates within the structural plane of a bedding rock slope, whose surface acts as a reflective interface, resulting in the amplification of vibrations on the slope surface. The existence of the structural plane also induces the transmission and reflection of stress waves. In the case of the rock slope with a bedding structural plane, a continuous series of reflection waves forms between the slope and the structural plane, exerting a significant influence on the dynamic response of the slope. The superimposed stress field at the discontinuity can alter the stability characteristics of the rock mass at the discontinuity, increasing the risk of sliding. However, the majority of existing studies primarily focus on the free surface of the slope on the stress waves while frequently neglecting the influence of the structure on the transmission and reflection of the stress waves. In addition, in the case of actual slopes, the characteristics of the rock mass medium also play a crucial role in influencing the amplitude of stress waves within the slope, which is rarely considered in the current research efforts. For example, Shi et al.,^17^ and Yang et al.,^18^ assumed that the rock mass behaved as a linear elastic body when examining the distribution of the superimposed stress field within the slope, without considering the attenuation effect of viscoelastic stress wave in the rock mass. For viscoelastic rock mass, Wang et al.,^19^ established the stress wave propagation equation based on Kelvin model, which allows for the theoretical calculation of amplitude attenuation and time delay in the process of wave propagation. Expanding on this framework, Chai et al.,^20^ analyzed the slope vibration caused by P-wave incidents on the viscoelastic rock slope featuring a structural plane, and thoroughly considered the velocity superposition of multiple stress waves on the slope. However, their study did not include an analysis of the stress change at the structural plane from the stress field, nor did it explore the stability of the upper rock mass of the structural plane.

In view of this, starting from the propagation process of stress wave, this paper will theoretically analyze the superimposed stress field induced by multiple reflected waves occurring between the slope and the structural plane of the bedding rock slope, so as to establish the sliding criterion of slope along the structural plane under dynamic load. Firstly, the propagation path of single stress wave incident on rock mass is analyzed; subsequently, based on the time-domain analysis recursive method (TDRM) and the basic law of stress wave propagation in viscoelastic media^21,22^, the calculation equation of superimposed velocity field at any point of the structural plane through different propagation paths is established; furthermore, considering the gravity effect of rock mass and the stress distribution characteristics of structural plane, the slope slip criterion is established. This study will contribute to the understanding of the propagation law of stress wave in rock mass with structural plane to a certain extent, and provide a theoretical basis for further analysis of the response and stability evaluation of rock slopes under dynamic loads, such as earthquakes and blasting events.

## Analysis of waves’ propagation paths in rock slope with bedding structure plane

**Fig. 1:**
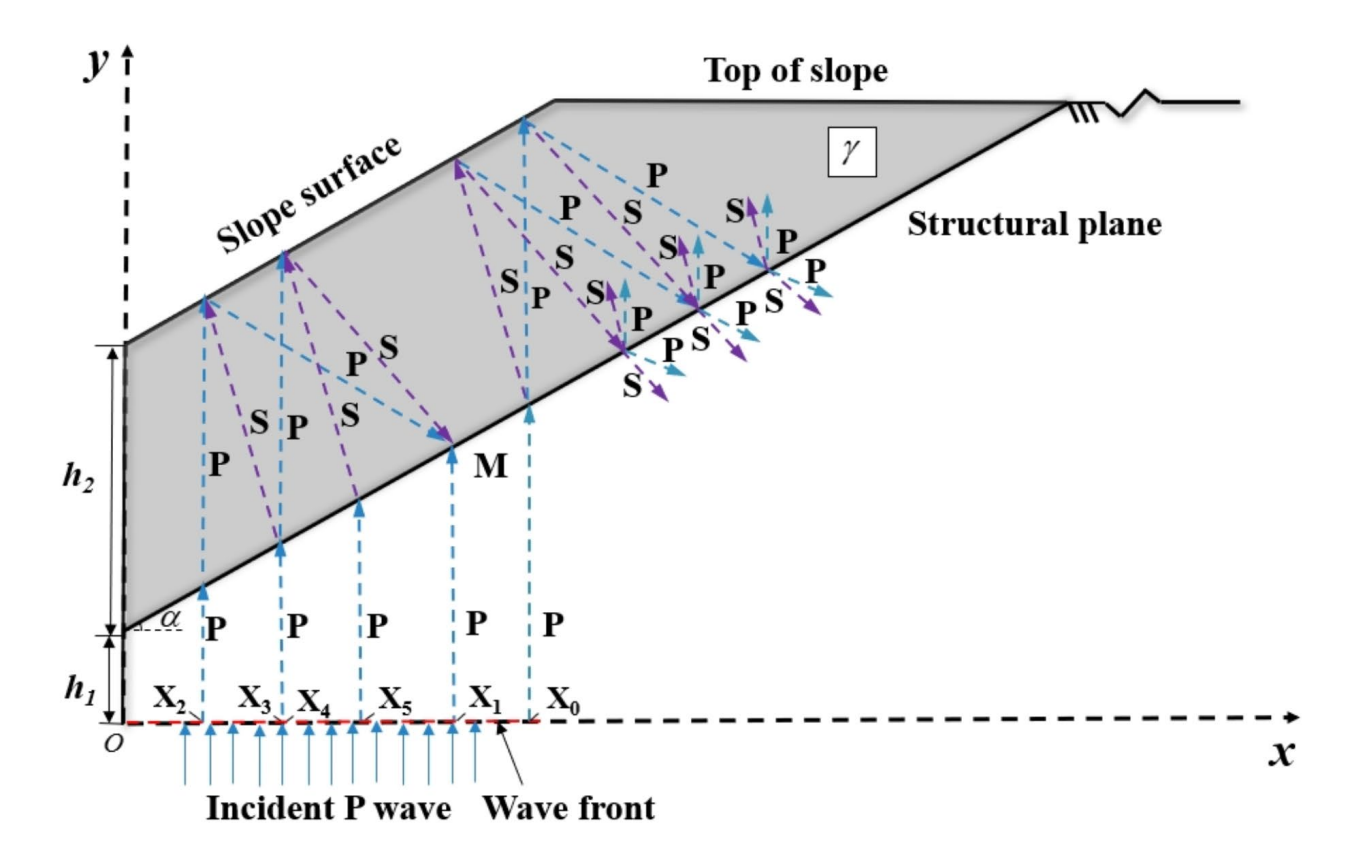
Schematic diagram of P wave propagation in rock slope with a bedding structure plane.

The calculation diagram of the stress field on both sides of the structural plane caused by P-wave incident on the rock slope with a bedding structural plane is shown in Fig. 1 The slope features a continuous structural plane that runs parallel to the slope. The angle between this structural plane and the horizontal direction is defined as *α*. It is assumed that the mass weight of the slope body is denoted as $\gamma$. The length of the structural plane is *l*, the height of the structure from the ground is *h*_*1*_, and the height of the structural plane from the slope is *h*_*2*_. According to the previous study^20^, it has been established that stress waves undergo multiple reflections between the structural plane and the slope surface, forming multiple reflected P- and S-waves. The superposition of each of these waves will cause the change of the stress field within the rock mass. Simultaneously, the slope block located above the structural plane may slide due to the combined effects of gravity on the rock mass. This issue is attributed to the stress distribution at the structural plane.

It is assumed that the initial wave front is parallel to the x-axis, that is, the incident wave is vertically incident to the slope body along the z-axis. The propagation path of stress waves within the rock slope with bedding structural plane is shown in Fig. 1P- and S-waves transmitted through the structural plane follow distinct paths, reaching various locations on the ground surface. These trajectories can be obtained according to the geometric relationships of each wave’s propagation path. The initial incident angles of transmitted P and S waves satisfy Snell’s law. For a more detailed analysis, please refer to the article^20^. The waves that reach the slope surface undergo reflection at the slope surface. These reflected P- and S-waves will propagate back into the slope body until reaching the structural plane, which leads to changes in the stress field at the structural plane. After that, a series of multiple reflected waves formed between the structural plane and the slope surface, which will cause the superposition of slope vibration response once reaching the slope surface. When these waves reach the structural plane, they result in the superposition of the stress field at the structural plane, subsequently affecting the stability of the slope.

When a half-sine initial wave *v*_*0*_, with an amplitude A and frequency *f* is introduced at position X_0_, the propagation distance *L*_0_, and propagation time *t*_0_ from the incident rock mass to the structural plane at *t* = 0 can be obtained according to the geometric relationship in Fig. 1.

1 $$v_{0}^{{\text{J}}}(t)=\exp \left( { - {a_{\text{p}}}{L_{\text{0}}}} \right) \cdot {v_{\text{0}}} \cdot \left( {t - {t_{\text{0}}}} \right)$$

Where $v_{0}^{{\text{J}}}(t)$ is the velocity when the incident P-wave reaches the structural plane, m/s; *a*_p_ is the viscoelastic coefficient.

$v_{0}^{{\text{J}}}(t)$ propagation in the structural plane generates transmitted P-and S-waves $v_{{{\text{Tp0}}}}^{{\text{J}}}$, $v_{{{\text{Ts0}}}}^{{\text{J}}}$, it can be calculated by using the TDRM of stress wave propagation at the structural plane in article^21^.

However $v_{{{\text{Tp0}}}}^{{\text{J}}}$, $v_{{{\text{Ts0}}}}^{{\text{J}}}$, (abbreviated as $v_{{{\text{Tm0}}}}^{{\text{J}}}$, m = p, s) will reach the slope after a certain distance ${L_{\text{m}}}$ and time ${t_{{\text{1m}}}}={L_{{\text{1m}}}}/{c_{\text{m}}}$, and the amplitude attenuation and time delay will also occur due to the viscoelasticity of rock mass in the process of propagation. The calculation equation is consistent with Eq. (1).

The slope surface will reflect the incident P-and S-waves, resulting in the formation of reflected P-and S-waves, respectively. These four waves will reach the structural plane and cause the superposition of stress fields. Concurrently, the structural plane reflects these four waves, giving rise to a total of eight reflected waves. These reflected waves undergo a sequence of continuous reflections between the structural plane and the slope surface, causing alterations in the stress field at the structural plane each time they reach it. The slip of the structural plane is intricately connected with the stress values at various points within the structural plane.

From the preceding research, it is evident that when waves transmitted from the slope and reflected by the structural plane reaches the slope again, there is a significant amplitude attenuation, and a noticeable time delay is observed. Therefore, the subsequent multiple reflection processes have little effect on the stress of the structural plane, which will not be considered in this paper. When analyzing the stress field at the structural plane, only the five wave propagation processes mentioned above are taken into account. According to Fig. 1, the stress wave reaching a point M on the structural plane is the result of the superposition of initial stress waves at different positions X_1_-X_5_ through different propagation paths.

## The stress caused by a single stress wave acting the structural plane

**Fig. 2:**
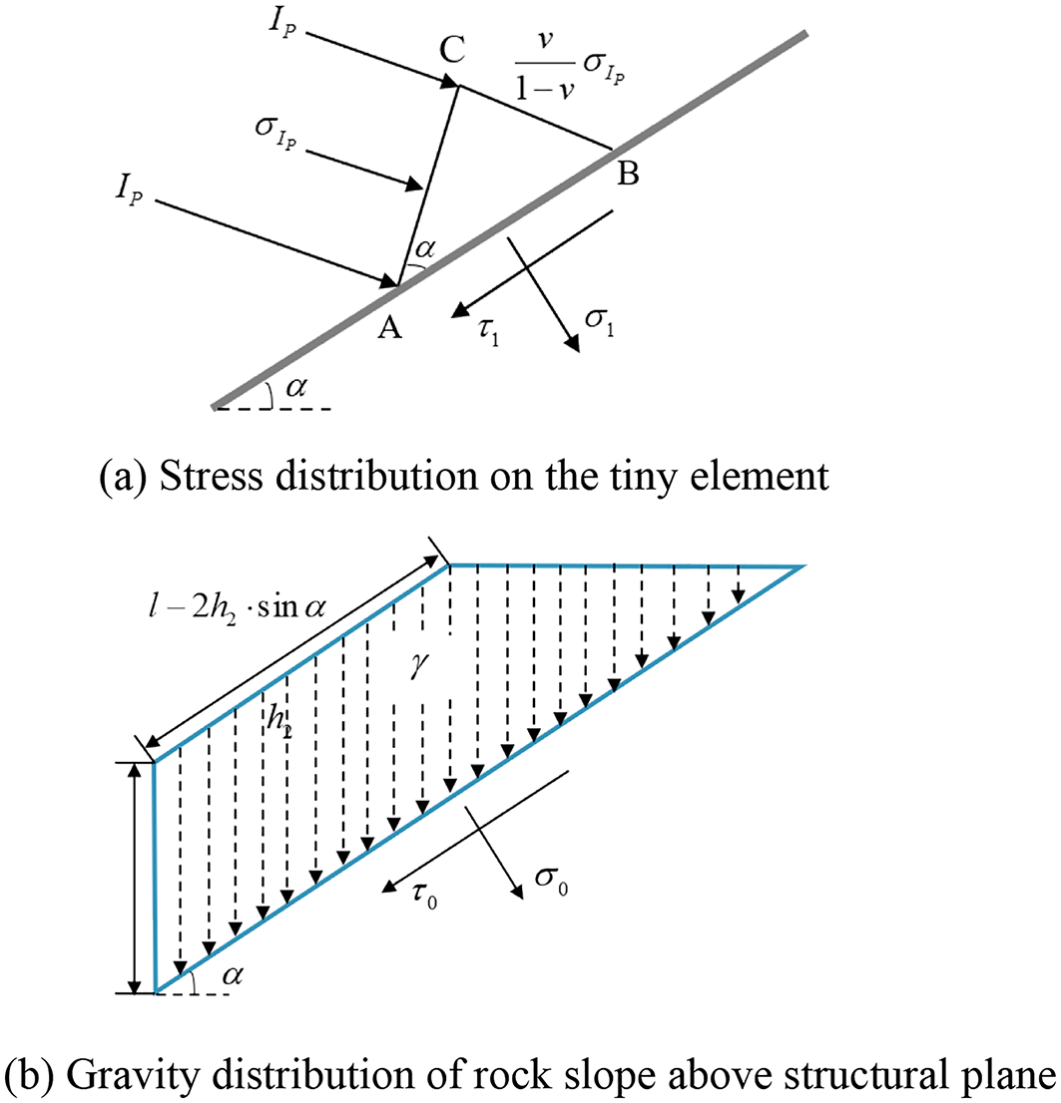
Schematic diagram of the small element body and gravity diagram of rock slope on structural plane.

When plane P-wave incident on the structural plane, the incident wave, transmitted P-waves, S-waves and reflected P-waves, S-waves will collectively form a tiny element with the structural plane and the wavefront. In paper of the TDRM^21^, the iterative equations for transmitted and reflected waves were derived through the balance relationship between the physical forces of each tiny element and the conservation of momentum on the wavefront. This paper maintains a consistent approach to analyze the stress field at the structural plane. For example, Fig. 2 (a) shows a tiny element composed of incident waves, where the line segment AC represents the wave front of the incident P wave, and the stress magnitude on the BC side is $\frac{v}{{1 - v}}{\sigma _{{\text{Ip}}}}$, where ${\sigma _{{\text{Ip}}}}$ is the normal stress of the incident P wave in front of the wave, *ν* is the Poisson’s ratio of intact rock.

In tiny element ABC, *σ*_1_ and *τ*_1_ represent the normal stress and shear stress on the left interface of the structural plane. Based on equilibrium conditions, it can be deduced that:

2 $${\sigma _1}={\sigma _{{\text{Ip}}}}\cos 2\beta ,{\tau _1}={\sigma _{{\text{Ip}}}}\sin 2\beta \operatorname{tg} \beta \operatorname{ctg} \alpha$$

Similarly, the stress on the structural plane caused by transmitted P, S waves, and reflected P, S waves *σ*_*i*_、*τ*_*i*_(*i* = 2 ~ 5)can be represented as:

3 $${\sigma _2}={\sigma _{{\text{Rp}}}}\cos 2\beta ,{\tau _2}= - {\sigma _{{\text{Rp}}}}\sin 2\beta \operatorname{tg} \beta \operatorname{ctg} \alpha$$

4 $${\sigma _3}= - {\tau _{{\text{Rs}}}}\sin 2\beta ,{\tau _3}= - {\tau _{{\text{Rs}}}}\cos 2\beta$$

5 $${\sigma _4}={\sigma _{{\text{Tp}}}}\cos 2\beta ,{\tau _4}={\sigma _{{\text{Tp}}}}\sin 2\beta \operatorname{tg} \beta \operatorname{ctg} \alpha$$

6 $${\sigma _5}= - {\tau _{{\text{Ts}}}}\sin 2\beta ,{\tau _5}={\tau _{{\text{Ts}}}}\cos 2\beta$$

When considering the gravity of the slope, the slope above the structural plane will exert stress on the structural plane, as shown in Fig. 2 (b). At this point, the stress on the tiny element satisfies:

7 $${\sigma _0}=\frac{{\gamma \cdot S}}{l} \cdot \cos \alpha ,{\tau _0}=\frac{{\gamma \cdot S}}{l} \cdot \sin \alpha$$

Where $S = \left({2L - h_{2}\sin \alpha - \frac{{{h_2}\, \text{cos}\, \alpha }}{{\tan \alpha }}} \right) \cdot \frac{{h_{2} \cdot \text{cos}\, \alpha }}{2}$ represents the volume of rock blocks on the left side of the structural plane within the plane; *h*_*2*_ is the vertical distance between the slope surface and the structural surface; *l* is the length of the structural plane in the slope body; *γ* is the weight of the rock mass.

According to the law of conservation of momentum on the wavefront, we can obtain the stress generated by stress waves at each element ${\sigma _{{\text{Ip}}}} = {z_{\text{p}}}{v_{{\text{Ip}}}}$, ${\sigma _{{\text{Rp}}}} = {z_{\text{p}}}{v_{{\text{Rp}}}}$, ${\tau _{{\text{Rs}}}} = {z_{\text{s}}}{v_{{\text{Rs}}}}$, ${\sigma _{{\text{Tp}}}} = {z_{\text{p}}}{v_{{\text{Tp}}}}$ and ${\tau _{{\text{Ts}}}} = {\text{-}}{z_{\text{s}}}{v_{{\text{Ts}}}}$, where ${v_{{\text{Ip}}}}$, ${v_{{\text{Rp}}}}$, ${v_{{\text{Tp}}}}$ are the particle velocities of incident P-wave, reflected P-wave, and transmitted P-wave, respectively; *v*_Rs_ and *v*_Ts_ represent the particle velocities of reflected and transmitted S-waves, respectively; The wave impedances are ${z_{\text{p}}}=\rho {c_{\text{p}}}$ and ${z_{\text{s}}}=\rho {c_{\text{s}}}$, where $\rho$ is the complete rock density; *c*_p_ and *c*_s_ represent the longitudinal and transverse wave velocities of intact rocks, respectively. Taking the rock block on the left side of the structural plane for further analysis, the stress on the left interface of the structural plane can be expressed as follows:

8 $${\sigma ^ - }={\sigma _0}+{\sigma _1}+{\sigma _2}+{\sigma _3}={\sigma _0}+{z_{\text{p}}}\cos 2\beta \cdot {v_{{\text{Ip}}}}+{z_{\text{p}}}\cos 2\beta \cdot {v_{{\text{Rp}}}} - {z_{\text{s}}}\sin 2\beta \cdot {v_{{\text{Rs}}}}$$

9 $${\tau ^ - }={\tau _0}+{\tau _1}+{\tau _2}+{\tau _3}={\tau _0}+{z_{\text{p}}}\sin 2\beta \operatorname{tg} \beta \operatorname{ctg} \alpha \cdot {v_{{\text{Ip}}}} - {z_{\text{p}}}\sin 2\beta \operatorname{tg} \beta \operatorname{ctg} \alpha \cdot {v_{{\text{Rp}}}} - {z_{\text{s}}}\cos 2\beta \cdot {v_{{\text{Rs}}}}$$

**Fig. 3:**
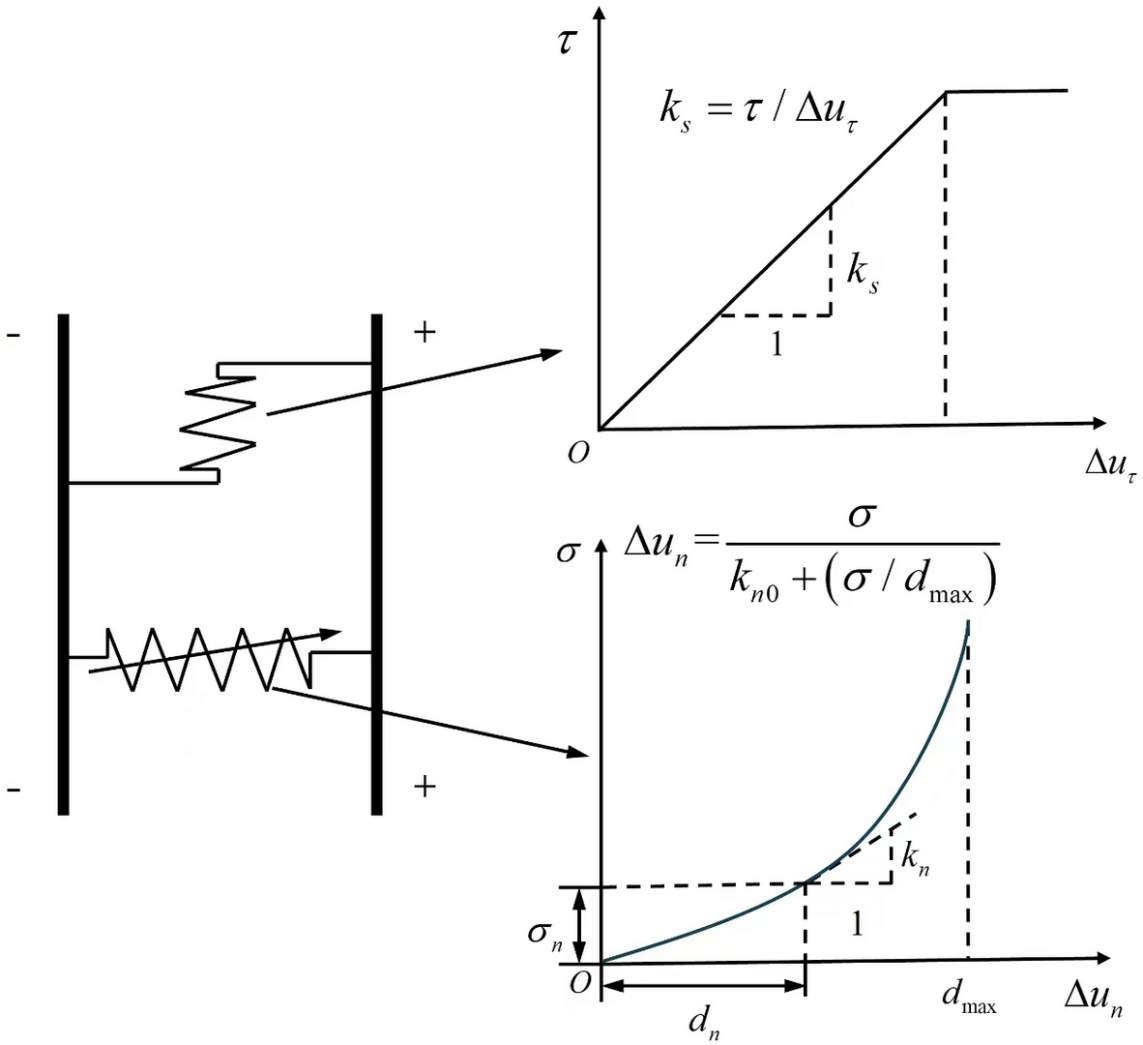
Schematic diagram of rock structural plane model.

The crucial aspect of the equation above lies in determining the particle velocities of reflected P- and S-waves, as well as transmitted P and S-waves. This can be resolved by utilizing the stress wave propagation equation incident upon a nonlinear structural, as discussed plane in paper^19^. Assuming that the rock mass structural plane exhibits a normal nonlinear BB model as shown in Fig. 3, where the normal deformation on the structural plane follows a hyperbolic BB model and the tangential direction is a linear elastic model. In Fig. 3, *k*_*s*_ signifies the tangential stiffness of the structural plane, Δ*u*_τ_ represents the shear displacement between the structural planes, and *τ* represents the tangential stress acting on the structural plane; Δ*u*_n_ represents the nornal displacement between the structural planes, σ_n_ represents compressive stress on both sides of a structural plane, *d*_*n*_ represents the closure quantity of structural plane,*k*_*n*_ is the normal stiffness of the structural plane, *k*_*n0*_ is the initial normal stiffness of the structural plane, *d*_max_ is the maximum allowable closure of the structural plane, and the propagation equation of stress waves can be expressed as:

10 $${\left[ {\begin{array}{*{20}{c}} {{v_{{\text{Rp}}}}} \\ {{v_{{\text{Rs}}}}} \end{array}} \right]_{(i)}}= - {B^{{\text{-1}}}}A{v_{{\text{Ip}}(i)}}+{B^{{\text{-1}}}}C{\left[ {\begin{array}{*{20}{c}} {{v_{{\text{Tp}}}}} \\ {{v_{{\text{Ts}}}}} \end{array}} \right]_{(i)}}$$

11 $${\left[ {\begin{array}{*{20}{l}} {{v_{{\text{Tp}}}}} \\ {{v_{{\text{Ts}}}}} \end{array}} \right]_{(i+1)}}={C^{{\text{-1}}}}{D_{(i)}}{v_{{\text{Ip}}(i)}}+{C^{{\text{-1}}}}{E_{(i)}}{\left[ {\begin{array}{*{20}{l}} {{v_{{\text{Rp}}}}} \\ {{v_{{\text{Rs}}}}} \end{array}} \right]_{(i)}}+{C^{{\text{-1}}}}{F_{(i)}}{\left[ {\begin{array}{*{20}{l}} {{v_{{\text{Tp}}}}} \\ {{v_{{\text{Ts}}}}} \end{array}} \right]_{(i)}}$$

Where:

12 $$A=\left[ {\begin{array}{*{20}{c}} {{z_{\text{p}}}\cos (2\beta )} \\ {{z_{\text{p}}}\sin (2\beta )\tan \beta /\tan \alpha } \end{array}} \right]$$

13 $$B=\left[ {\begin{array}{*{20}{c}} {{z_{\text{p}}}\cos (2\beta )}&{ - {z_{\text{s}}}\sin (2\beta )} \\ { - {z_{\text{p}}}\sin (2\beta )\tan \beta /\tan \alpha }&{ - {z_{\text{s}}}\cos (2\beta )} \end{array}} \right]$$

14 $$C=\left[ {\begin{array}{*{20}{c}} {{z_{\text{p}}}\cos (2\beta )}&{{z_{\text{s}}}\sin (2\beta )} \\ {{z_{\text{p}}}\sin (2\beta )\tan \beta /\tan \alpha }&{ - {z_{\text{s}}}\cos (2\beta )} \end{array}} \right]$$

15 $${\sigma _{(i)}}={z_{\text{p}}}\cos (2\beta ) \cdot {v_{{\text{Ip}}(i)}}+{z_{\text{p}}}\cos (2\beta ) \cdot {v_{{\text{Rp}}(i)}} - {z_{\text{s}}}\sin (2\beta ) \cdot {v_{{\text{Rs}}(i)}}$$

16 $${k_{{\text{n}}(i)}}={\left( {{k_{{\text{n0}}}}+{\sigma _{({\text{1}})}}/{d_{{\text{max}}}}} \right)^{\text{2}}}/{k_{{\text{n0}}}}$$

17 $${D_{(i)}}=\left[ {\begin{array}{*{20}{c}} {{k_{{\text{n}}(i)}}\Delta t\cos \alpha } \\ {{k_{\text{s}}}\Delta t\sin \alpha } \end{array}} \right]$$

18 $${E_{(i)}}=\left[ {\begin{array}{*{20}{c}} { - {k_{{\text{n}}(i)}}\Delta t\cos \alpha }&{{k_{{\text{n}}(i)}}\Delta t\sin \beta } \\ {{k_{\text{s}}}\Delta t\sin \alpha }&{{k_{\text{s}}}\Delta t\cos \beta } \end{array}} \right]$$

19 $${F_{(i)}}=\left[ {\begin{array}{*{20}{c}} { - {k_{{\text{n}}(i)}}\Delta t\cos \alpha +{z_{\text{p}}}\cos (2\beta )}&{ - {k_{{\text{n}}(i)}}\Delta t\sin \alpha +{z_{\text{s}}}\sin (2\beta )} \\ { - {k_{\text{s}}}\Delta t\sin \alpha +{z_{\text{p}}}\sin (2\beta ) \cdot \tan \beta /\tan \alpha }&{{k_{\text{s}}}\Delta t\cos \beta - {z_{\text{s}}}\cos (2\beta )} \end{array}} \right]$$

## Stress field analysis and slip criterion at the structural plane

### The stress field at the structural plane

Section 1 describes the multiple transmission and reflection process of a single P-wave incident through the structural plane and slope. Consequently, when a group of plane P-waves incident on a slope with structural plane, each incident P-wave will propagate according to the above law. Therefore, at any point on the structural plane, multiple beams of waves incident from different positions at the bottom of the slope will arrive sequentially after one or more reflections, as shown in Fig. 1. At point M of the structural plane, a P-wave (1 beam in total) directly arrives the structural plane from X_1_. Additionally, there are also P-and S-waves (4 beams in total) that are incident from X_2,_ X_3_, X_4_ and X_5_ after undergoing multiple reflections between the structural plane and slope surface.

**Fig. 4:**
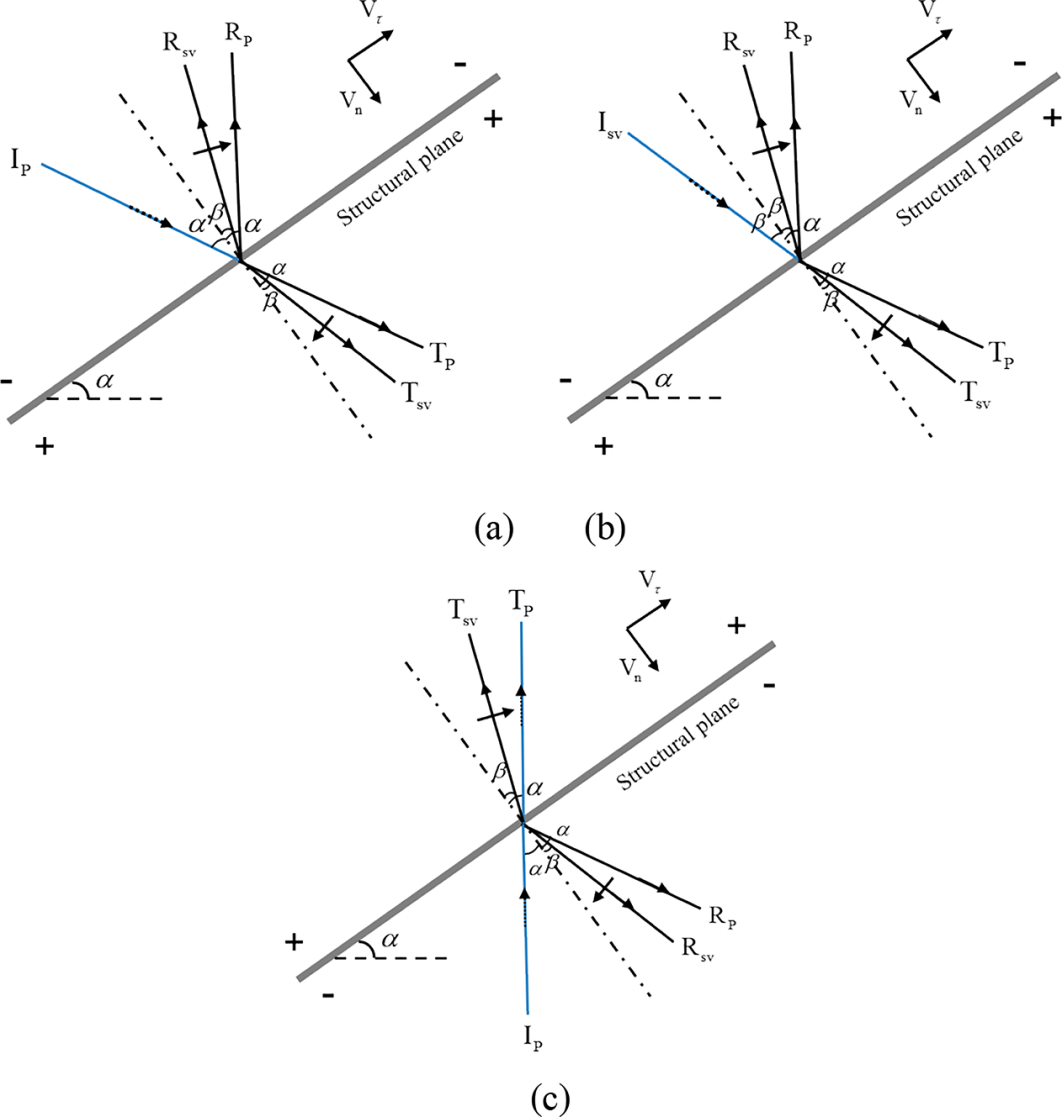
Diagram of interaction between three types of waves and structural plane.

Based on Eqs. (8)-(9) and Fig. 1, the stress field at a monitoring point on the structural plane is primarily a consequence of two factors: the stress field caused by the velocity of the stress wave and the stress field attributed to the weight of the slope block. The stress field caused by the stress wave can be categorized into three types: the stress field caused by the direct incident P-wave at the slope bottom, the stress field generated by the reflected P-wave on the slope surface, and the stress field produced by the reflected S-wave on the slope surface. Figure 4 provides a schematic representation of the interaction between these three wave types and the structural plane.

As shown in Fig. 4(a), the transmission and reflection of stress wave on the structural plane can be obtained as follows: any beam of P wave $v_{{{\text{Ip}}}}^{{\text{f}}}(t)$ reflected from slope surface *α*. The normal vibration ${v_{{\text{n1}}}}(t)$ and tangential vibration $v_{t1}(t)$ caused by the incident angle on the structural plane can be expressed as:

20 $${\left[ {\begin{array}{*{20}{l}} {{v_{{\text{n1}}}}} \\ {{v_{{\text{t1}}}}} \end{array}} \right]_{(i)}}=\left[ {\begin{array}{*{20}{c}} {\cos \alpha } \\ {\sin \alpha } \end{array}} \right] \cdot v_{{{\text{Ip}}(i)}}^{{\text{f}}}+\left[ {\begin{array}{*{20}{c}} { - \cos \alpha }&{\sin \beta } \\ {\sin \alpha }&{\cos \beta } \end{array}} \right] \cdot {\left[ {\begin{array}{*{20}{l}} {{v_{{\text{Rp}}}}} \\ {{v_{{\text{Rs}}}}} \end{array}} \right]_{(i)}}+\left[ {\begin{array}{*{20}{c}} {\cos \alpha }&{\sin \beta } \\ {\sin \alpha }&{ - \cos \beta } \end{array}} \right] \cdot {\left[ {\begin{array}{*{20}{l}} {{v_{{\text{Tp}}}}} \\ {{v_{{\text{Ts}}}}} \end{array}} \right]_{(i)}}$$

By substituting Eq. (20) into the relationship between particle vibration velocity and stress caused by stress wave, it can be obtained:

21 $${\left[ {\begin{array}{*{20}{l}} {{\sigma _{{\text{n1}}}}} \\ {{\tau _{{\text{t1}}}}} \end{array}} \right]_{(i)}}=\left[ {\begin{array}{*{20}{c}} {\cos \alpha } \\ {\sin \alpha } \end{array}} \right] \cdot v_{{{\text{Ip}}(i)}}^{{\text{f}}} \cdot {z_{\text{p}}}+\left[ {\begin{array}{*{20}{c}} { - \cos \alpha }&{\sin \beta } \\ {\sin \alpha }&{\cos \beta } \end{array}} \right] \cdot {\left[ {\begin{array}{*{20}{l}} {{v_{{\text{Rp}}}} \cdot {z_{\text{p}}}} \\ {{v_{{\text{Rs}}}} \cdot \left( { - {z_{\text{s}}}} \right)} \end{array}} \right]_{(i)}}+\left[ {\begin{array}{*{20}{c}} {\cos \alpha }&{\sin \beta } \\ {\sin \alpha }&{ - \cos \beta } \end{array}} \right] \cdot {\left[ {\begin{array}{*{20}{l}} {{v_{{\text{Tp}}}} \cdot {z_{\text{p}}}} \\ {\left. {{v_{{\text{Ts}}}} \cdot ( - {z_{\text{s}}}} \right)} \end{array}} \right]_{(i)}}$$

Where: ${\sigma _{{\text{n1}}}}$ direction is vertical and the face down is positive, ${\tau _{{\text{t1}}}}$ direction is positive along the structural face.

**Fig. 5:**
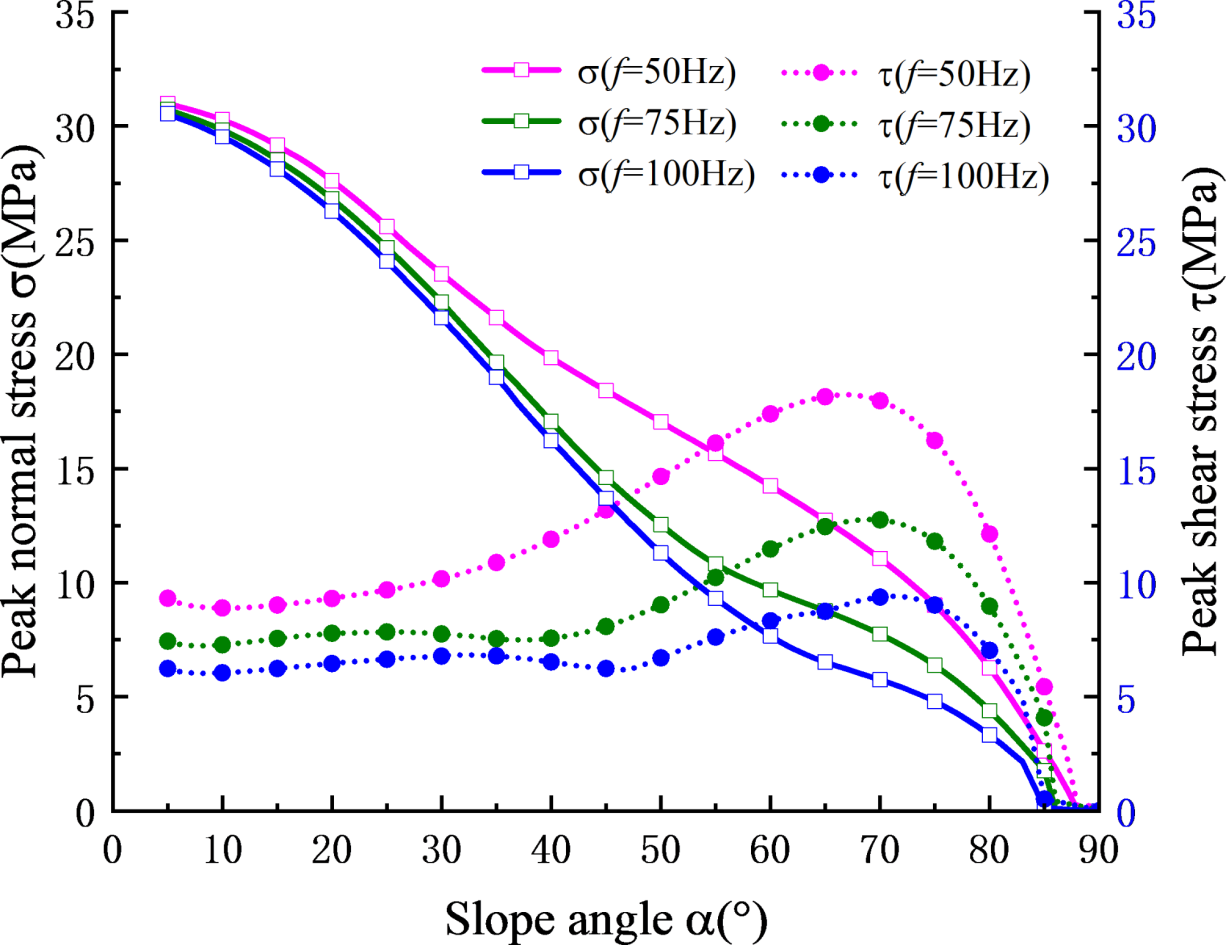
Relationship between stress peak value and slope angle at three frequencies of incident wave.

Similarly, as shown in Fig. 5 (b), the stress field caused by the incident angle$\beta$of any beam of S-waves reflected from the slope surface on the structural plane can be expressed as:

22 $$\begin{aligned} {\left[ {\begin{array}{*{20}{l}} {{\sigma _{{\text{n2}}}}} \\ {{\tau _{{\text{t2}}}}} \end{array}} \right]_{(i)}}&=\left[ {\begin{array}{*{20}{c}} {\sin \beta } \\ { - \cos \beta } \end{array}} \right] \cdot v_{{{\text{Is}}(i)}}^{{\text{f}}} \cdot \left( { - {z_{\text{s}}}} \right)+\left[ {\begin{array}{*{20}{c}} { - \cos \alpha }&{\sin \beta } \\ {\sin \alpha }&{\cos \beta } \end{array}} \right] \cdot {\left[ {\begin{array}{*{20}{l}} {{v_{{\text{Rp}}}} \cdot {z_{\text{p}}}} \\ {{v_{{\text{Rs}}}} \cdot \left( { - {z_{\text{s}}}} \right)} \end{array}} \right]_{(i)}} \hfill \\&\quad +\left[ {\begin{array}{*{20}{c}} {\cos \alpha }&{\sin \beta } \\ {\sin \alpha }&{ - \cos \beta } \end{array}} \right] \cdot {\left[ {\begin{array}{*{20}{l}} {{v_{{\text{Tp}}}} \cdot {z_{\text{p}}}} \\ {{v_{{\text{Ts}}}} \cdot \left( { - {z_{\text{s}}}} \right)} \end{array}} \right]_{(i)}} \hfill \\ \end{aligned}$$

As shown in Fig. 5 (c), the incident P-wave $v_{{IP}}^{g}(t)$ at the bottom of any beam slope. The stress field caused by the incident angle *α* on the structural plane can be expressed as:

23 $$\begin{aligned} {\left[ \begin{gathered} {\sigma _{{\text{n3}}}} \hfill \\ {\tau_{{\text{t3}}}} \hfill \\ \end{gathered} \right]_{(i)}}&=\left[ {\begin{array}{*{20}{c}} { - \cos \alpha } \\ {\sin \alpha } \end{array}} \right] \cdot v^{{\text{g}}}_{{\text{Ip}}{(i)}} \cdot {z_{\text{p}}}+\left[ {\begin{array}{*{20}{c}} {\cos \alpha }&{\sin \beta } \\ {\sin \alpha }&{ - \cos \beta } \end{array}} \right] \cdot {\left[ \begin{gathered} {v_{{\text{Rp}}}} \cdot {z_{\text{p}}} \hfill \\ {v_{{\text{Rs}}}} \cdot -{z_{\text{s}}} \hfill \\ \end{gathered} \right]_{(i)}} \hfill \\&\quad +\left[ {\begin{array}{*{20}{c}} { - \cos \alpha }& {\sin \beta } \\ {\sin \alpha } &{\cos \beta } \end{array}} \right] \cdot {\left[ \begin{gathered} {v_{{\text{Tp}}}} \cdot {z_{\text{p}}} \hfill \\ {v_{{\text{Ts}}}} \cdot -{z_{\text{s}}} \hfill \\ \end{gathered} \right]_{(i)}} \hfill \\ \end{aligned}$$

As presented in Fig. 1, five stress waves reaching any point ${X^{\prime}_0}$ on the structural plane will change the velocity field at that point, and then affect the stress field. Therefore, the stress fields at ${X^{\prime}_0}$ are respectively the sum of the stress fields caused by the five waves (gravity stress should also be considered), namely:

24 $$\left\{ {\begin{array}{*{20}{c}} {\sigma _{{{{{\text{X}^{\prime}}}_{\text{0}}}}}^{{\text{n}}}(t)=\sum\limits_{{j=1}}^{5} {{\sigma _{{\text{n}}j}}(t)+{\sigma _0}} } \\ {\tau _{{{{{\text{X}^{\prime}}}_{\text{0}}}}}^{{\text{t}}}(t)=\sum\limits_{{j=1}}^{5} {{\tau _{{\text{t}}j}}(t) - {\tau _0}} } \end{array}} \right.$$

In order to solve the normal stress field ${\sigma _{{\text{n}}j}}(t)$ and tangential stress field ${\tau _{{\text{t}}j}}(t)$ in the above equation, it is only necessary to comprehensively analyze the propagation equation and process of single wave, and obtain the equation and incidence angle of the initial stress wave incident at any position when it reaches the structural plane, and then calculate the partial stress field from Eqs. (20)-(21).

### Slip criterion at the structural plane

Generally, rock masses tend to slide more easily along bedding structural planes. In the case of relatively stable rock masses, the component force generated by gravity from the overlying rock mass on the bedding structural plane is typically lower than the sliding strength of the structural plane itself. When stress waves impact the rock mass, the multiple reflections of stress waves result in stress superposition at the structural plane. The bedding structural plane provides a natural sliding surface for the rock mass, and the strength of the filled layer is low, under the combined action of the structural plane stress caused by gravity and stress wave propagation, when the sliding stress component exceeds the sliding strength of the structural plane, it is easy to cause the sliding of the upper rock mass on the structural plane, threatening the stability of the slope. As depicted in Fig. 1, the rock mass at the upper left of the structural plane may experience sliding. Assuming that the shear strength of the structural plane satisfies the Coulomb slip model, the sliding strength at any point on the structural plane, denoted as *τ*_*f*_, can be expressed as:

25 $${\tau _{\text{f}}}(t)=\sigma _{{{{\text{X}}_{\text{0}}}}}^{{\text{n}}}(t) \cdot \tan \varphi +c$$

Where: *φ* is the sliding internal friction angle of structural plane; *c* is the cohesion between structural plane fillings; $\sigma _{{{{\text{X}}_{\text{0}}}}}^{{\text{n}}}(t)$ is the normal stress at any point ${X^{\prime}_0}$ at the structural plane, which can be obtained from Eq. (24). Due to the fluctuation characteristics of stress waves, the normal stress changes in real time, causing the anti-sliding strength at each point to vary over time. The anti-sliding force of the entire structural plane at any moment is the summation of the anti-sliding strength values of all particles. Consequently, the slip condition on the structural plane can be expressed as the difference between the total sliding force T(*t*) and the anti-sliding force T_*f*_(t), namely:

26 $$F(t)={\rm T}(t) - {{\rm T}_f}(t)=\int_{0}^{l} {\tau \left( {x,t} \right)} - \int_{0}^{l} {{\tau _f}\left( {x,t} \right)} =\sum {\tau _{{{{{\text{X}^{\prime}}}_{\text{0}}}}}^{{\text{t}}}(t) \cdot \Delta l - } \sum {{\tau _f}_{{{{{\text{X}^{\prime}}}_{\text{0}}}}}^{{\text{t}}}(t) \cdot \Delta l}$$

When F(*t*) ≥ 0, it is considered that the sliding force exceeds the anti-sliding force, resulting in sliding failure. Where, it can be calculated from Eq. (24); Δ*l* represents the width of each discrete point along the direction of the structural plane. The above results can be obtained through a semi-analytic solution implemented in MATLAB, with time (t) being discretized during the calculation process. The relationship between Δ*x* and Δ*t* and the relationship between Δ*l* and Δ*t* can be deduced from the geometric considerations and the wave propagation velocity.

Stability coefficient is commonly employed to assess the stability of the slope. In this paper, the minimum value of slope stability coefficient, *K*_*smin*_, at each moment of stress waves action time is taken as the criterion for slippage determination, as follows:

27 $${K_{s\hbox{min} }}=\hbox{min} \frac{{{{\rm T}_f}(t)}}{{{\rm T}(t)}}=\hbox{min} \frac{{\sum {{\tau _f}_{{{{{\text{X}^{\prime}}}_{\text{0}}}}}^{{\text{t}}}(t) \cdot \Delta l} }}{{\sum {\tau _{{{{{\text{X}^{\prime}}}_{\text{0}}}}}^{{\text{t}}}(t) \cdot \Delta l} }}$$

If *K*_*smin*_<1, it indicates that sliding failure has occurred along the structural plane of the slope. To provide a more comprehensive analysis of the impact of stress wave propagation on slope sliding characteristics, the slope stability coefficient *K*_*s0*_ without stress waves action is calculated for comparison, as follows:

28 $${K_{s0}}=\frac{{{{\rm T}_{f0}}}}{{{{\rm T}_0}}}=\frac{{{\sigma _0}\tan \varphi +c \cdot l}}{{{\tau _0}}}$$

Where *σ*_0_ and *τ*_0_ are obtained from Eq. (7), combined with Eq. (7), the above equation can be rewritten as:

29 $${K_{s0}}=\cot \alpha \tan \varphi +\frac{{c \cdot l}}{{{\tau _0}}}$$

When the angle of the structural plane is less than the internal friction angle of the structural plane, *k*_*s0*_ remains consistently greater than 1, indicating the structural plane’s stability. If the inclination angle of the structural plane exceeds the internal friction angle, the cohesion of the structural plane becomes a significant factor. Under the influence of incident stress waves, the stress superposition from multiple reflected waves changes the sliding force and anti-sliding force at the structural plane. Therefore, it is very important to study the conditions and factors of slope sliding under incident stress waves.

## Parameter analysis and discussion

To investigate the stress wave propagation and associated slip risk in bedding rock slopes, this section will analyze the absolute peak stress at the monitoring point at the structural plane and the stability coefficient of the slope respectively from two aspects of rock slope properties and structural plane parameters. The specific parameters include slope angle $\alpha$, monitoring point location ${X^{\prime}_0}$, vertical distance of potential sliding mass ${h_2}$ and initial normal stiffness of structural plane ${K_{n0}}$.

The theoretical analysis model of the slope is consistent with Fig. 1. A half-sin P-wave is input at the bottom of the model, expressed as ${V_0}=A\sin (2\pi ft)(0 \leqslant t \leqslant 1/2f)$, with the amplitude of *A* = 1 m/s. The maximum closure of the structural plane is taken as 1mm^23,24^. The stress calculation method at the structural plane has been thoroughly elaborated in Sect. 3. Unless stated otherwise, the basic calculation parameters of the rock slope with a bedding structural plane are list in Table 1.

**Table 1:** Basic calculation parameters of rock mass.

S-wave velocity (m/s)	*P*-wave Velocity (m/s)	Rock density (kg/m^3^)	Initial normal stiffness (GPa/m)	Initial shear stiffness (GPa/m)	Friction angle (°)	d_max_ (mm)
5380	2650	2650	1.3	0.7	45	1

### Influence of different slope angles on peak stress

In order to assess the influence of different slope angles on the peak stress of the structural plane, the calculation placed the monitoring point at ${X^{\prime}_0}$= 30 m. The vertical height of the slope foot was taken as *h*_1_ = 10 m, *h*_2_ = 20 m, the initial normal stiffness of the structural plane, *k*_n0,_ was taken as 1.3 GPa/m, and the tangential stiffness, *k*_s0,_ was taken as 0.7 GPa/m. The relationship curves of the absolute value of the peak normal stress and peak shear stress at the monitoring point with respect to the slope angle are shown in Fig. 5.

It can be seen from Fig. 5 that the absolute peak normal stress at the monitoring points decreases with the slope angle increases, while the absolute peak shear stress initially increases and then decreases as the slope angle increases. The primary reason for the change in the normal stress peak value is the gradual reduction of the vertical component of the total stress at the monitoring point with increasing slope angle, leading to a decrease in the normal stress peak value. The main reason for the change of the peak shear stress is that with the continuous increase in the slope angle, the transmission attenuation effect of the rock slope structure on the stress wave continues to increase. This results in the tangential component of the total stress value at the monitoring point increasing less than the decrease in shear stress due to the attenuation effect. Therefore, the peak shear stress curve begins to decline at around 70°, and this downward trend gradually increases. This also shows that the increase of slope angle has a significant effect on the attenuation of stress wave.

The peak values of normal stress and shear stress at the monitoring point generated by low frequency incident waves are significantly higher than those produced by high frequency incident waves, which also confirms the nature of structural plane to pass the low frequency and resist high frequency. According to the theoretical research results of Li et al.^23^, when the incident wave frequency gradually increases, the stress wave gradually attenuates and the stress value at the structural plane gradually decreases, which verifies the research results of this subsection.

### Influence of different monitoring points on peak stress

**Fig. 6:**
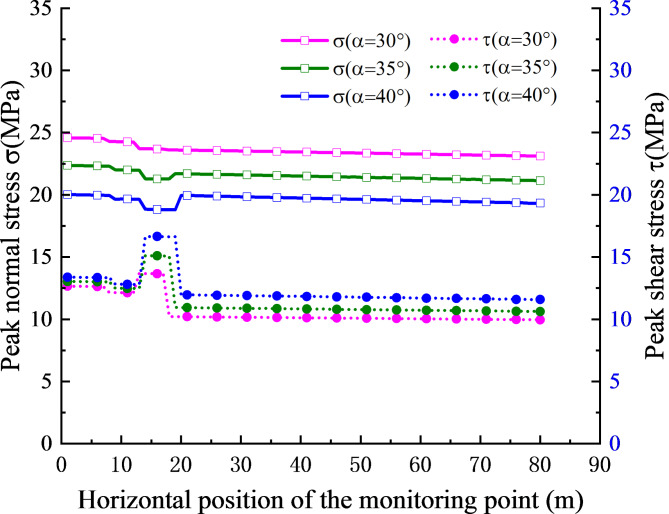
Relationship between stress peak value and monitoring point location.

To investigate the impact of the horizontal position of different monitoring points on the peak stress at the slope structural plane, this section considered the vertical height of the slope foot as *h*_1_ = 10 m, *h*_2_ = 20 m, with an incident wave frequency of *f* = 50 Hz. The relationship curves between the absolute value of the peak normal stress and the peak shear stress and the horizontal position of the monitoring point are shown in Fig. 6.

As depicted in Fig. 6, the peak values of normal stress and shear stress after the horizontal position of the monitoring point${X^{\prime}_0} \geqslant$20m gradually decrease and stabilize, with relatively minor fluctuations. The fluctuation of this part primarily result from the attenuation of the stress waves due to the increasing propagation distance as the monitoring point’s horizontal position rises. At the horizontal position of the monitoring point 0 m$<{X^{\prime}_0} \leqslant$20 m, a slight fluctuation is observed in the peak of normal stress. For instance, at an incident wave frequency of 50 Hz, the slope angle of 30^o^ and the monitoring point ${X^{\prime}_0}$ at 1 m, the normal stress is 24.59 MPa, and the shear stress is 12.66 MPa. In this scenario, only the stress wave incident from X_1_ in Fig. 1 can reach the monitoring point. Based on the geometric relationship of the wave propagation paths shown in Fig. 1, when the coordinates of the monitoring point are substantial, except for the wave directly incident to the point, other multiple reflected waves cannot reach the structural plane due to angular deflection in their propagation path or transmission into the rock mass after reaching the top of the slope, so the stress superposition effect is not obvious. As the horizontal position of the monitoring point varies from 0 m to 20 m (0 m$<{X^{\prime}_0} \leqslant$20 m), the incident waves at X_5_, X_3_, X_4_ and X_2_ reach the monitoring point in turn, which leads to the stress at the structural plane becoming complex, for example, when the incident wave frequency is 50 Hz, the slope angle is 40^o^ and the monitoring point ${X^{\prime}_0}$ is 19 m and 20 m respectively, the arrival of incident wave at X_2_ causes the shear stress to decreases from 16.63 MPa to 11.97 MPa, resulting in significant fluctuations in the peak shear stress. Consequently, the peak stress in this interval is affected by many factors, such as the attenuation of waves, the increase in the number of waves, and the peak spacing of different waves.

At the same monitoring point, the peak value of normal stress decreases as the slope angle increases, and the peak value of shear stress increases with the increase in the slope angle, in accordance with the trends discussed in Sect. 5.1.

### Influence of incident wave frequency

**Fig. 7:**
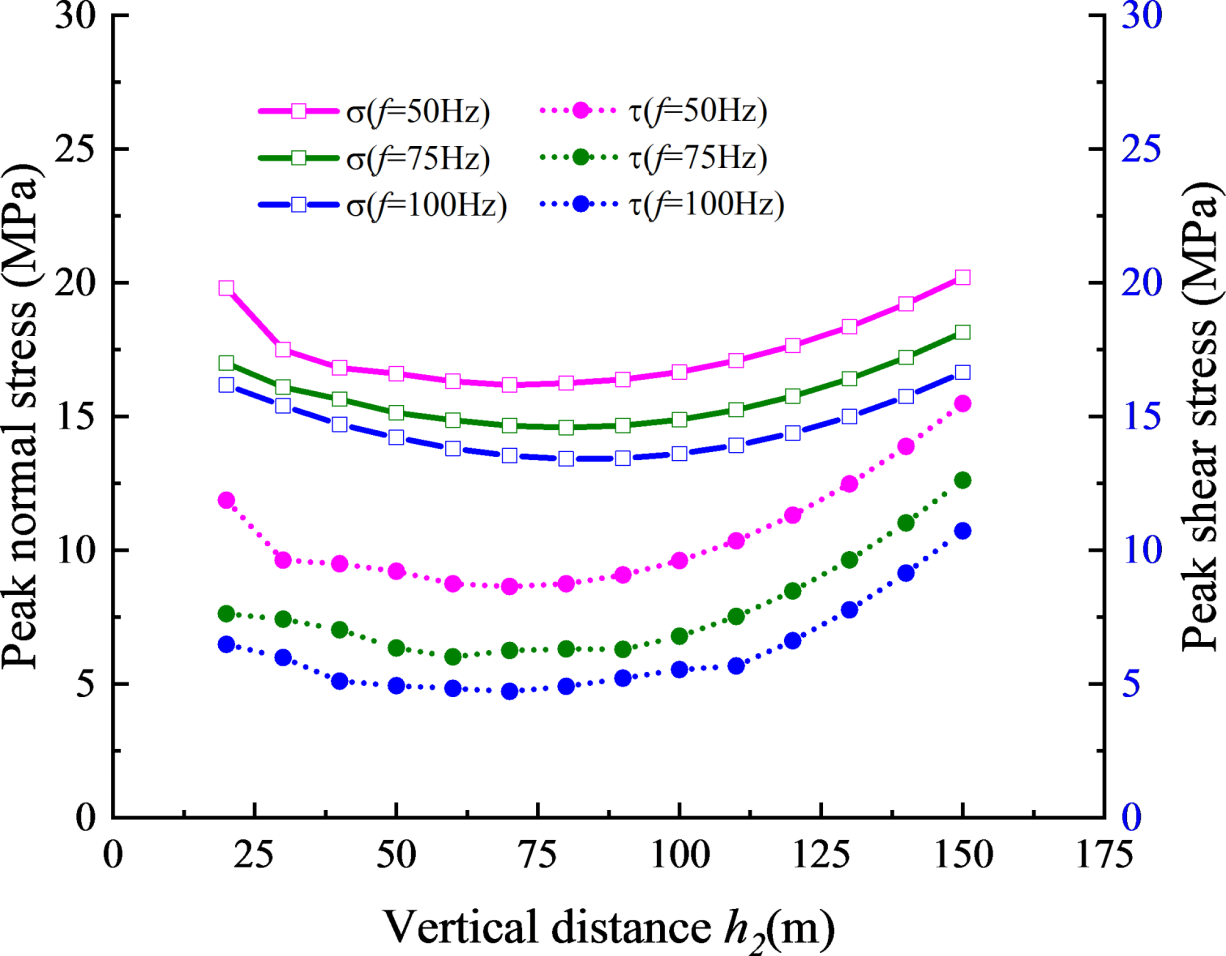
Relationship between stress peak value and vertical distance of slope toe.

It can be seen from Fig. 1, the alteration in the vertical distance between the structural plane and the rock slope influences the volume of the potential sliding body located above the structural plane. Additionally, it affects the propagation distance of stress waves within the rock mass. In this section, the intersection of the horizontal line at the top of the vertical distance *h*_2_ and the structural plane is selected as the monitoring point (location ${X^{\prime}_0}{\text{=}}\sqrt 3 {h_2}$), the slope angle *α* is 30^o^, and the vertical distance between the structural plane and the ground ${h_1}$ is 10 m. The curves of stress peak change caused by variations in the vertical distance between the structural plane and the slope are shown in Fig. 7 for three incident frequencies.

Figure 7 demonstrates that the peak normal stress and shear stress at the monitoring point on the structural plane exhibit a consistent variation trend under different frequencies. Both stress parameters initially exhibit a gradual decrease, followed by a gradual increase with an increase in slope height. For the change curve of peak stress value at a specific monitoring point under a single frequency, its change is mainly affected by three factors: the increase of gravity stress due to the rise in slope height, wave attenuation resulting from the increased stress wave propagation distance in the rock mass, and the change of superposition section of each beam stress wave caused by the increase in slope height.

**Fig. 8:**
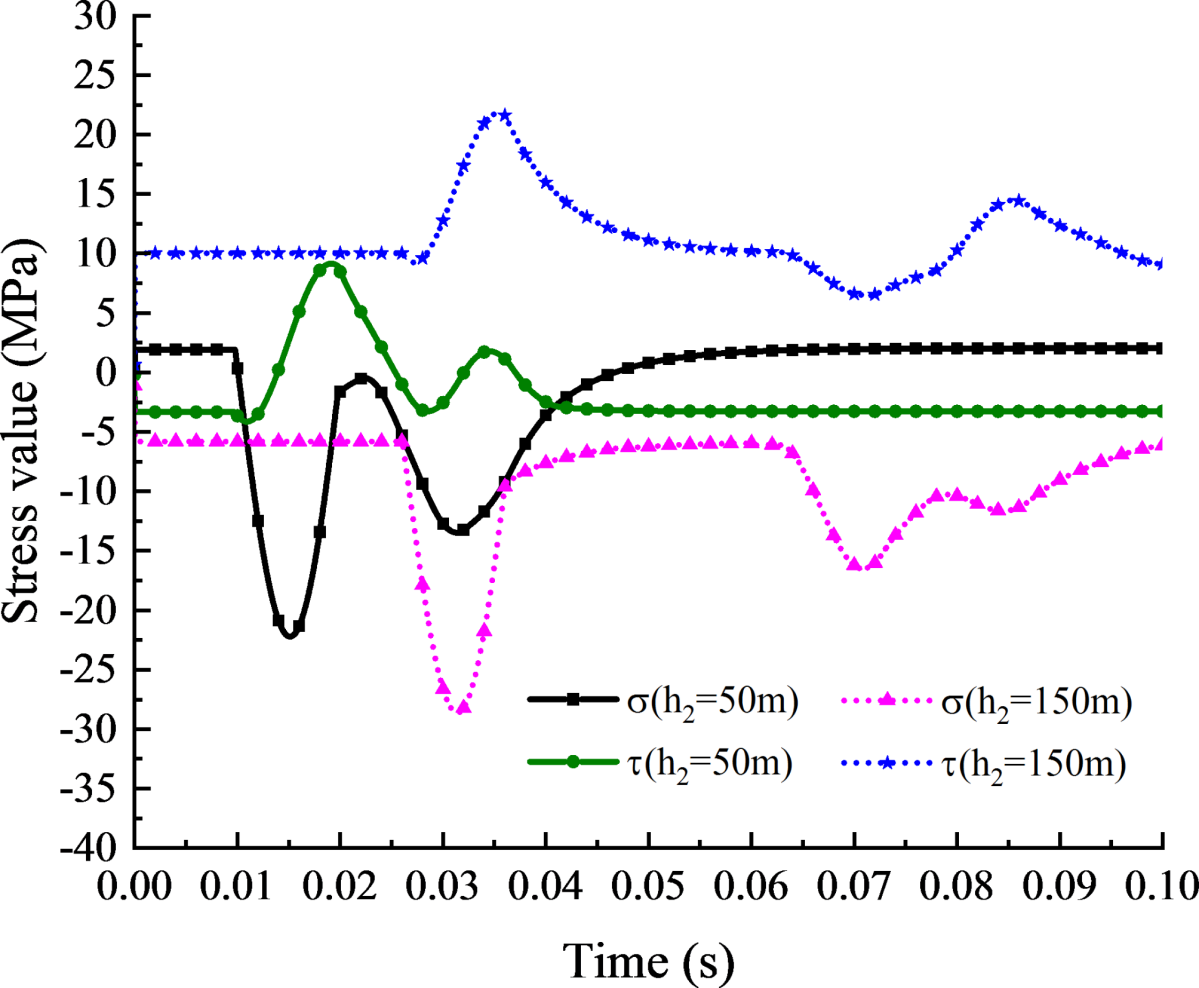
The curve of stress value at h2 = 50 m and h2 = 150 m with time.

To identify the key factors affecting stress peak variation, the stress value variation curves with time at vertical distances of *h*_2_ = 50 m and *h*_2_ = 150 m (*f* = 50 Hz) are compared and analyzed, as shown in Fig. 8 It can be found that when *t* = 0 s, the peak normal stress and shear stress at the vertical distance of 50 m are 0.38 MPa and 0.22 MPa, while the peak normal stress and shear stress at the vertical distance of 150 m are 1.13 MPa and − 0.65 MPa, respectively. Therefore, the minimal change in the peak stress values is attributed to the increase of self-weight stress due to the rise in slope height. Additionally, the wave attenuation caused by slope height is relatively small. Consequently, the primary reason for the change of the peak stress value caused by the vertical distance is that it indirectly changes the time of the superimposed waves reaches a specific monitoring point, thus changing the amplification effect of their superimposition. With the increase of the vertical distance of the rock slope, the stress value at the structural plane first increases and then decreases, which is the same as the test trend of Singh R et al.^11^.

### Influence of initial normal stiffness of structural plane on peak stress

This section primarily studies the influence of structural stiffness change of structural plane on the peak stress at the structural plane. Take dimensionless normal stiffness ${K_{\text{n}}}={k_{{\text{n0}}}}/\left( {{z_{\text{p}}}\omega } \right)$, where $\omega =2\pi f$. Since it is impossible for the initial normal and tangential stiffness of the structural plane to maintain independent changes, a general stiffness value of the structural plane is assumed as ${K_{\text{s}}}/{K_{\text{n}}}=0.5$. The tangential stiffness of structural plane is then calculated according to the equation: ${k_{{\text{s0}}}}={K_{\text{n}}} \cdot \left( {{z_s}\omega } \right)$. The slope angle and incident wave frequency are taken as 30° and 50 Hz respectively.

**Fig. 9:**
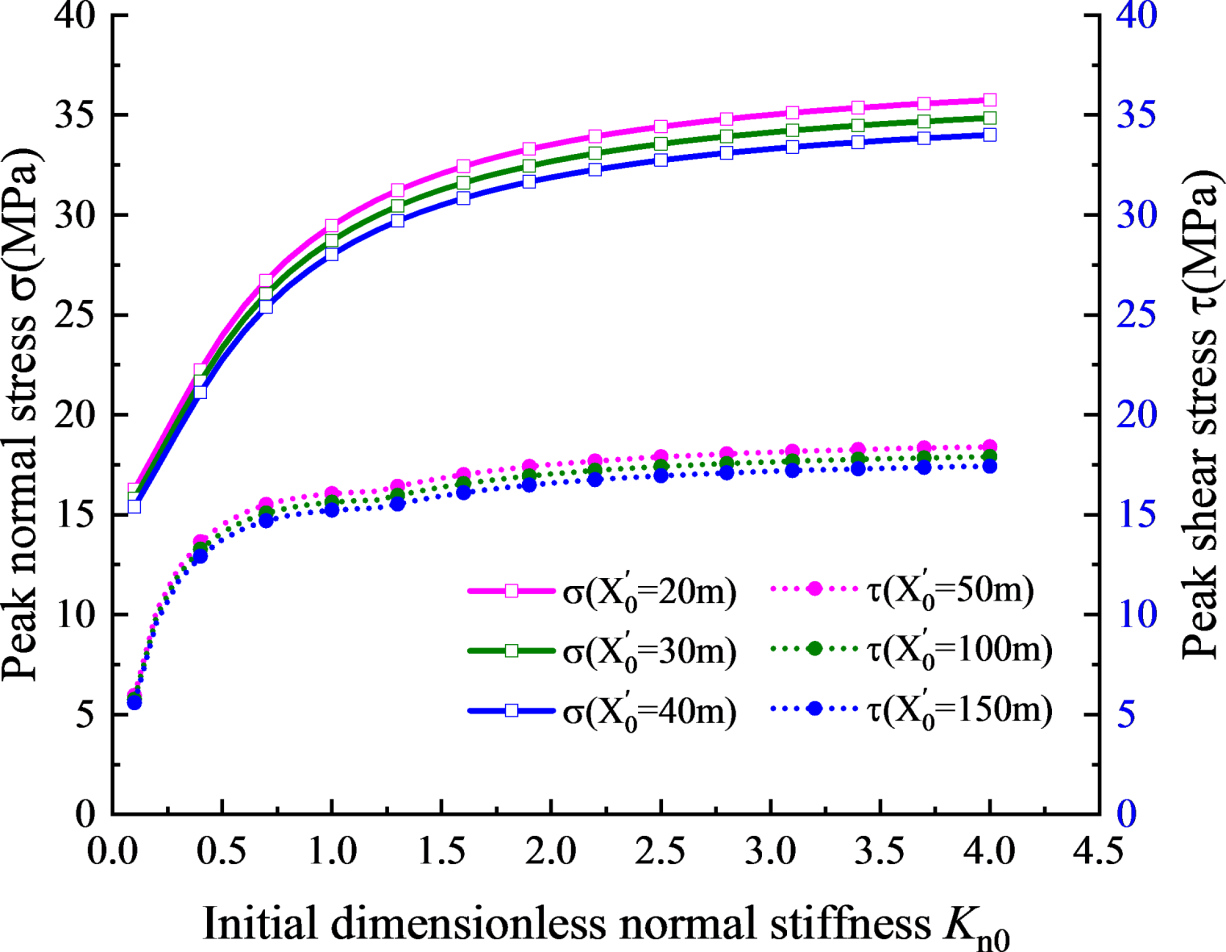
Relationship between stress peak value and normal stiffness.

As can be seen from Fig. 9, both the peak values of normal stress and shear stress exhibit a pattern of rapid increase and then slowly increase with the increase of normal stiffness, indicating a significant correlation between the peak value of stress and stiffness. At ${K_{\text{n}}}>1.5$, the peak value of stress increased slowly and gradually become stable. It can be inferred that with the increase of the initial normal stiffness of the structural plane, the transmission ability of the rock slope structure towards stress waves gradually increases, while the reflection ability gradually weakens. Consequently, the stress value at the monitoring point of the structural plane also gradually increases. With a continued increase in the initial normal stiffness, the transmission capacity of the structural plane for stress waves slowly increases until all the energy of the incident waves is transmitted to the transmission wave, resulting in that the peak stress at the monitoring point of the structural plane increases slowly and gradually tend to be stable. According to the theoretical research results of Chai et al.^21^, with the increase of the initial normal stiffness of the structural plane, the stress values at the structural plane increase rapidly and then tend to be stable, which proves the feasibility of the theoretical method used in this paper.

### Slip analysis of structural plane

**Fig. 10:**
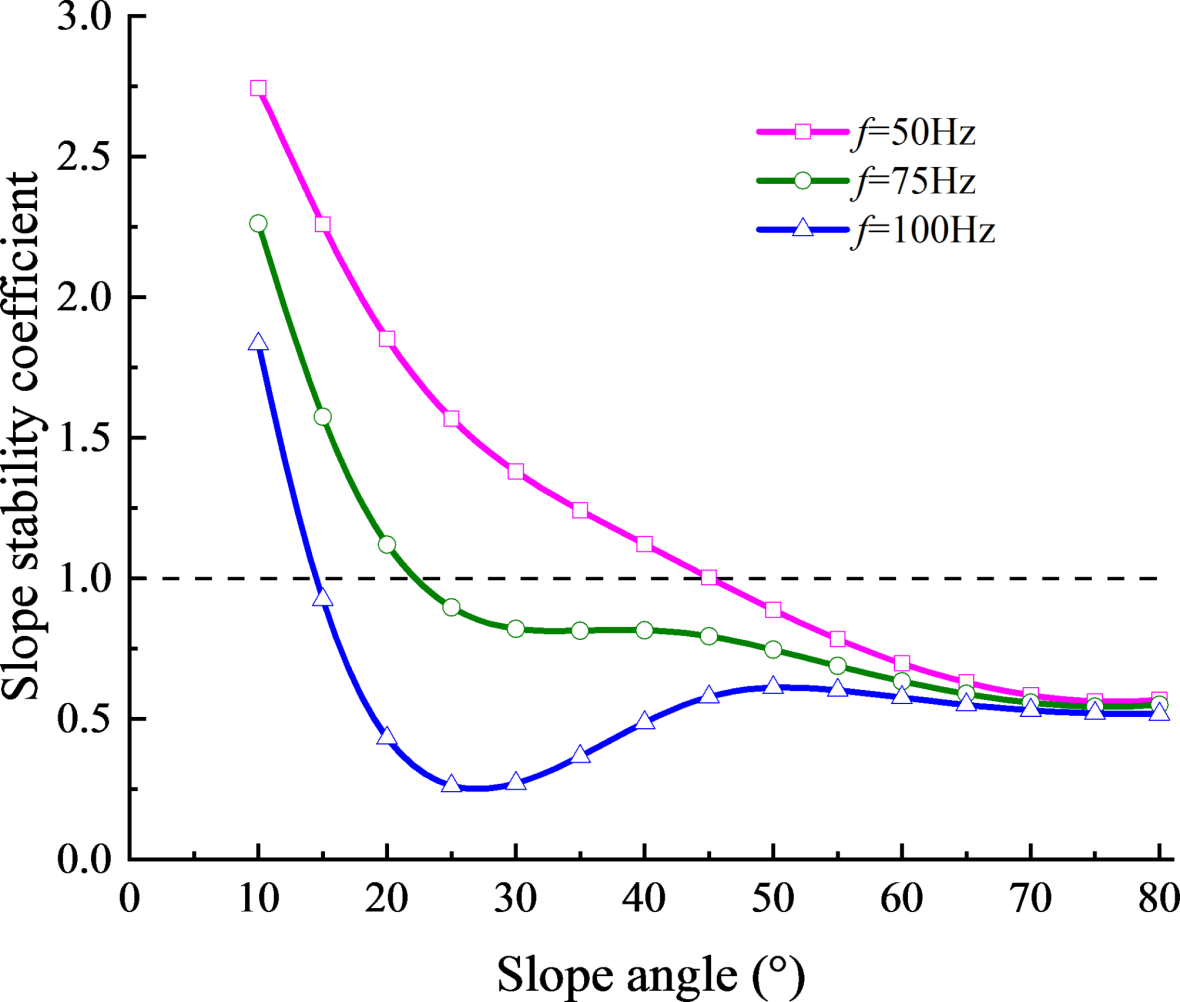
Relationship between slope stability factor and slope angle at three frequencies of incident wave.

Due to the existence of the structural plane in rock slope, slope stability coefficients were used for slip analysis. The slip criteria for slopes have been explained in Sect. 4.2. This section assumes that the shear strength of the structural plane satisfies the Coulomb slip model, with the slope stability coefficient as the primary research focus. The previous findings show that the change of the slope angle, the distance between the structural plane and the slope surface, and the initial normal stiffness have a significant impact on the stress at the structural plane. Therefore, this section analyzes the effects of different slope angles, the distance between the structural plane and the slope surface, and different initial normal stiffness on the minimum value *K*_*smin*_ of the slope stability coefficient. Following previous research^6^, when the cohesion “c” is greater than 0.22 MPa, the rock on both sides of the structural plane is well combined with the structural plane, and the rock mass on the upper part of the structural plane will not slide under the disturbance of environmental and human factors, excluding the influence of other factors on the slope stability coefficient in the parameter analysis below. Therefore, the cohesion “c” of structural plane is set to a constant value of 0.35 MPa. The relationship between the slope stability coefficient and the slope angle under three incident wave frequencies is shown in Fig. 10.

From Fig. 10, it can be seen that within the range of ${K_{s\hbox{min} }} \geqslant 1$, at a specific incident frequency, the slope stability coefficient gradually decreases with the increase of slope angle. When the incident frequency is set to 50 Hz, the minimum stability coefficient of the slope gradually decreases with the increase of the slope angle, primarily due to the gravity factor after the slope angle increases. Once the slope angle is greater than 45°, the sliding force exceeds the anti-sliding force, resulting in an unstable state where *K*_*smin*_ is less than 1. When the frequency increases to 75 Hz and 100 Hz, the slope slide occurs at around 23° and 15°, respectively, demonstrating the influence of multiple reflected waves. According to Fig. 5, due to the effect of stress waves, the normal stress gradually decreases with the slope angle, while the shear stress value decreases only when the slope angle exceeds 70°. The superposition of stress results in an unstable state of the slope at a small slope angle.

**Fig. 11:**
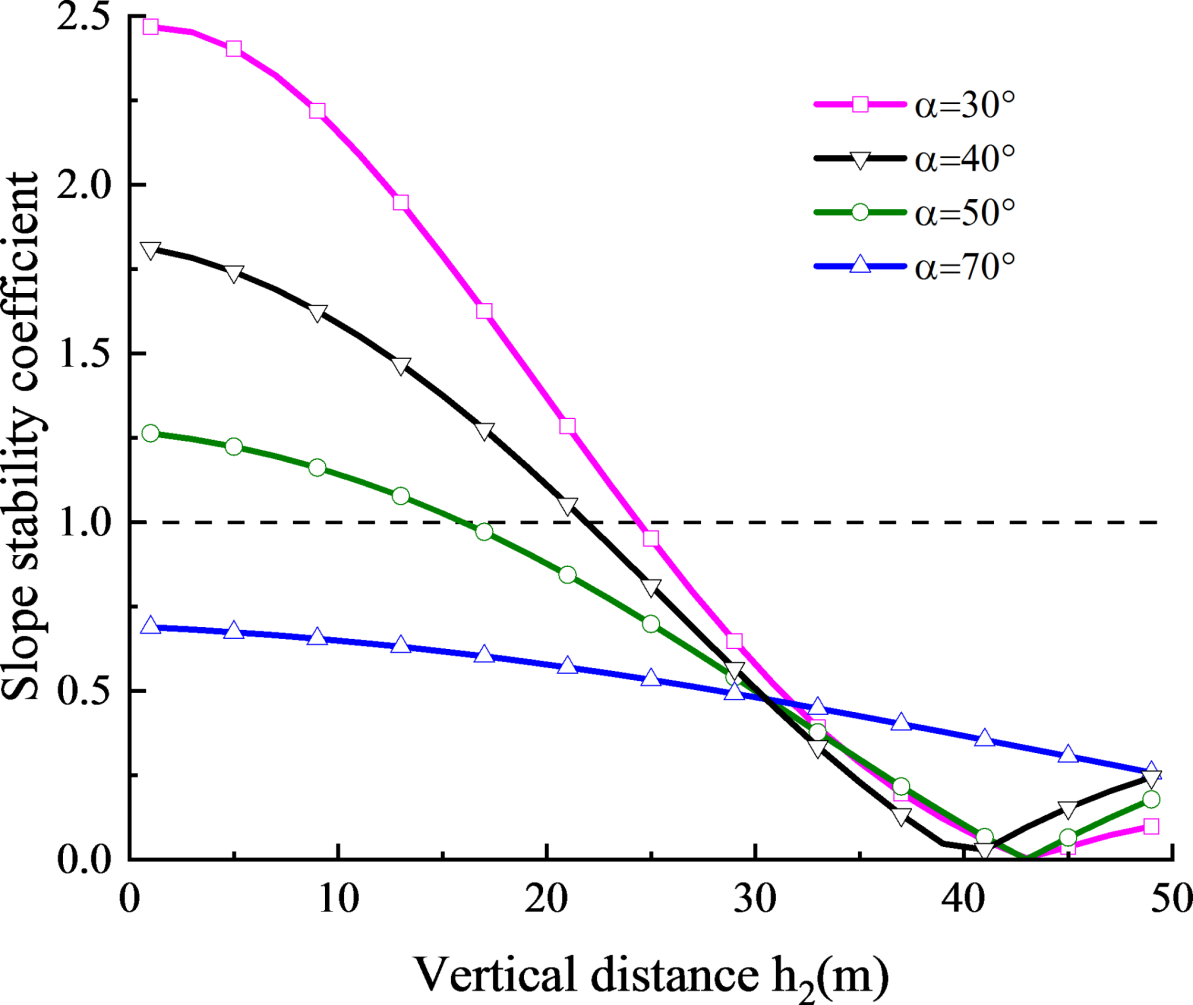
Relationship between slope stability coefficient and vertical distance of slope toe at four slope angles.

The variation of slope stability coefficient with the vertical distance from the structural plane to the top of the slope under four slope angles is shown in Fig. 11, with a constant frequency of 50hz. Within the range of ${K_{s\hbox{min} }} \geqslant 1$, the slope stability coefficient gradually decreases with an increase in vertical distance. In other words, when the slope angle is small, the slope begins to slipping as the vertical distance increases. For example, at a slope angle of 30°, sliding initiates at a vertical distance of 25 m. Similarly, at a slope angle of 50°, sliding occurs at a vertical distance of 16 m. With the increase of slope angle, the whole slope stability coefficient curve with slope angle of 70° remains below 1. This implies that when the slope reaches a certain angle, under the action of stress waves, the anti-sliding force and cohesion at the slope structural plane are less than the sliding force of the slope, and therefore the slope is always in the dangerous stage of sliding.

Within the range of ${K_{s\hbox{min} }}<1$, as the slope initiates sliding, there are many factors that affect the sliding force and anti-sliding force at the structural plane. For instance, cohesion and sliding friction will change along with the sliding during the sliding process. Seed^25^ also pointed out that when the slope stability coefficient falls below 1, the slope is not necessarily unstable and may be stabilized again after sliding for a certain distance. Consequently, this section focuses on the analysis of the conditions for the sliding of a rock slope with a structural plane and the influence of the slope angle and the vertical distance from the slope to the slope on the stability coefficient. The analysis of slope condition after sliding will not be further discussed here.

**Fig. 12:**
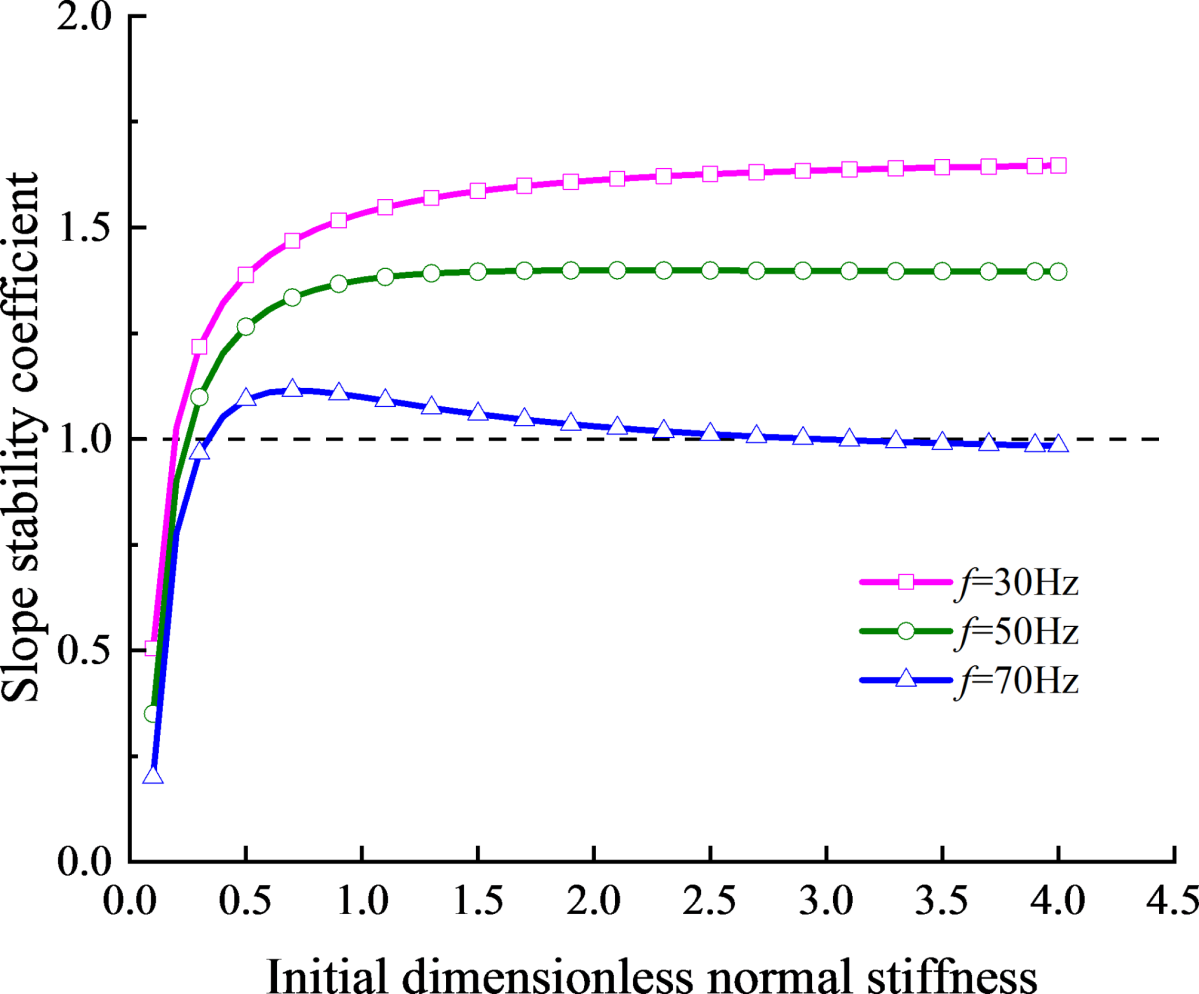
Relationship between slope stability coefficient and normal stiffness at three frequencies of incident wave.

Figure 12 presents the relationship between the slope stability coefficient and the initial normal stiffness under three incident wave frequencies, with the slope angle set at 30°. The results show that the slope stability coefficient initially increases rapidly with higher initial normal stiffness, stabilizing after reaching a certain value. However, it decreases with increased incident wave frequency and initial normal stiffness. Under the same initial normal stiffness, the slope stability coefficient for high frequency incident waves consistently remains lower than that for low frequency incident waves.

The increase of the initial normal stiffness within the range of ${K_n}<0.6$ is beneficial to improve the stability performance and isolation effect of the slope. With the continuous increase of the initial normal stiffness, the slope stability coefficient tends to be stabilize and remains above 1, indicating that the larger initial normal stiffness has a reduced impact on slope stability. However, under the action of high-frequency incident waves, the continuous increase of the initial normal stiffness leads to the continuous increase of the transmitted energy when the incident wave passes through the structural plane, which makes the slope stability coefficient decrease. When the slope stability coefficient falls below 1, the slope becomes prone to sliding.

## Conclusions

In this paper, the stress field and slope stability coefficient of a slope with a structural plane was analyzed. Through parameter analysis, the influence degree of various factors on the peak normal stress and shear stress of the structural plane was studied, and the factors causing slope slip were explored through the stability coefficient. The following conclusions were reached:

(1) The stress field at the structural plane of rock slope is generated by the superposition of the gravity stress of rock mass and multiple reflected waves. Stress waves and their multiple reflections significantly increase the risk of rock mass sliding on the structural plane of bedding rock slopes.

(2)The parameter study reveal that the slope angle, the horizontal position of monitoring point, the vertical distance from the structural plane to the slope surface and the initial normal stiffness of the structural plane all influence the stress field at the structural plane. The results show that the absolute value of the maximum stress at the structural plane is greatly affected by the initial stiffness and slope angle, and is less affected by the horizontal position of the monitoring point and the vertical distance from the structural plane to the slope surface.

(3) The influence laws of slope angle, vertical distance from structural plane to slope surface and initial normal stiffness of structural plane on slope stability coefficient are analyzed. When the stability coefficient of the slope is greater than 1, the slope stability coefficient decreases with the increase of slope angle and vertical distance from structural plane to slope surface; When the stability coefficient of the slope is below 1, the upper slope of the structural plane will lose stability, resulting in landslide, collapse and other destructive phenomena.

(4) With the increase of the initial normal stiffness, the slope stability coefficient increases rapidly, effectively enhancing slope stability. However, as the frequency increases, the energy of reflected waves becomes greater, leading to increased tensile stress and a gradual decrease in the slope stability coefficient. This decrease may cause the slope stability coefficient to fall below 1, indicating instability in the rock slope.

## Data Availability

The datasets used and/or analysed during the current study available from the corresponding author on reasonable request.
